# CHOICE international survey: Clusters of allergen immunotherapy prescription from French and Spanish cohorts

**DOI:** 10.1016/j.waojou.2023.100791

**Published:** 2023-07-01

**Authors:** Pablo Rodriguez del Rio, Davide Caimmi, Pilar Rico Nieto, Carmen Vidal, Carmen Moreno, Maria Teresa González-Fernández, Margarita Tomás-Pérez, Ana Beristain, Isa Bosse, Hoai Bich Trinh, Thomas B. Casale, Pascal Demoly, Moises A. Calderon, the CHOICE Survey Team

**Affiliations:** a Allergy Section, Hospital Infantil Universitario Nino Jesus, Madrid, Spain; b Allergy Unit, Department Respiratory Medicine and Allergy, Hôpital Arnaud de Villeneuve, University Hospital of Montpellier and IDESP, UMR UA11 Univ. Montpellier - INSERM, Montpellier, France; c Instituto de Medicina Molecular Aplicada, Facultad de Medicina San Pablo CEU Madrid, Spain; d Allergy Department, Faculty of Medicine USC and Complejo Hospitalario Universitario de Santiago, Santiago de Compostela, Spain; e Hospital Reina Sofia, Instituto Maimonides de Investigación Biomédica de Córdoba, Red ARADyAL, Cordoba, Spain; f Allergy Department, Hospital Universitario La Paz, Spain; g Allergy Section, Hospital Universitario Central de Asturias Oviedo, Spain; h Allergology Practice, La Rochelle, France; i Private Practice, Olivet, France; j Morsani College of Medicine, University of South Florida, Tampa, FL 33612, USA; k Imperial College London, U.K. and Faculty of Medicine, University of Costa Rica, Spain

**Keywords:** Allergen immunotherapy, Sublingual administration, Subcutaneous injection, Prescription, Rhinitis, Asthma, Cluster analysis

## Abstract

**Background:**

There is no description of the drivers of prescription for allergen immunotherapy (AIT) for respiratory allergic diseases.

**Methods:**

A prospective, multicentre, observational, non-interventional real-life study was performed in France and Spain for 20 months. Data were gathered using 2 different questionnaires, anonymously collected in an online platform. No names of AIT products were recorded. Multivariate analysis and unsupervised cluster analysis were performed.

**Results:**

One hundred and three physicians (50.5% from Spain and 49.5% from France) reported 1735 patients (433 in France and 1302 in Spain), 47.9% males, 64.8% adults with a mean age 26.2 years old. They suffered from allergic rhinitis (99%), allergic conjunctivitis (70.4%), allergic asthma (51.8%), atopic dermatitis (13.9%), and food allergy (9.9%). A clustering analysis based on 13 predefined relevant variables for AIT-prescription identified 5 different clusters, each of them including information regarding doctor's profile and patient demographics, baseline disease characteristics, and main AIT indication: 1) Looking at the future: focusing on asthma prevention (n = 355), 2) Efficacy after discontinuation of AIT (n = 293), 3) Fighting severe allergic disease (n = 322), 4) Looking at the present, facing current symptoms (n = 265) and 5) Doctor's own clinical experience (n = 500). Each one of these clusters have specific patients' and doctors' characteristics, representing distinctive AIT prescription drivers.

**Conclusion:**

Using data-driven analysis, we identified for the first time some reasons and patterns of AIT prescriptions in real-life clinical settings. There is no uniform indication for prescribing AIT, which varies amongst patients and doctors with multiple but specific drivers, taking into account several relevant parameters.

## Introduction

Over the last decades, the efficacy and safety profile of allergen immunotherapy (AIT), including both the subcutaneous (SCIT) and sublingual (SLIT) routes, have been thoroughly documented in different double-blind placebo-controlled randomized clinical trials (DBPC RCT)^1^, ^2^, ^3^ with highly selected patients and in real-life studies.^4^, ^5^, ^6^, ^7^ These evidence-based data have encouraged the current use of AIT worldwide and have also granted their inclusion in international guidelines such as Allergic Rhinitis and its Impact on Asthma (ARIA) for allergic rhinitis (AR)^8^ and Global INitiative for Asthma (GINA) for allergic asthma (AA).^9^

In this process, several different clinical parameters and outcomes have been evaluated; however, the identification of “drivers of prescription” is still lacking. These drivers of prescription represent the clinical criteria and context i) to prescribe AIT by the treating specialists and ii) to accept AIT treatment by the patients themselves. Considering the current use of AIT under the concept of “personalized medicine”,^10^^,^^11^ the identification of drivers of AIT prescription used in routine clinical practice in real life is extremely relevant, whilst remaining an unmet need.

Therefore, we designed an international academic project called “CHOICE: Criteria Used by Health Professionals on the Selection of Allergen Immunotherapy in Real Clinical Practice: an international e-survey”, which aims to identify the drivers for prescription when AIT is established as a new prescription to treat respiratory diseases caused by IgE-dependent-hypersensitivity to aeroallergens.

CHOICE is currently under development in 6 different areas of the world, including 22 countries. In this first publication we are presenting initial results, data obtained from the cohort of doctors and patients from France and Spain.

## Methods

### Study design

This was a prospective, multi-center, international, observational study. Data were collected during 20 months from real-life clinical settings. The full method's description is published elsewhere.^12^

### Population

#### Participating patients

Patients' inclusion criteria were: adults and children, males and females, with IgE-mediated pollen, house dust mites, animal dander and/or molds respiratory allergy who had just initiated AIT, either SCIT, SLIT-drops, or SLIT-tablets, according to standards of practice. Patients’ exclusion criteria were: patients who refused to participate and patients receiving food immunotherapy or hymenoptera venom immunotherapy.

### Survey questionnaires

This is a web-based survey (e-survey) in which 2 electronic questionnaires written in English were used: i) 1 questionnaire per participating doctor (Doctors' questionnaire, DQ) with 24 questions, taking 4–5 min to be completed and ii) 1 questionnaire per each patient (Patient's questionnaire, PQ) completed also by the participant doctor regarding each included patient starting with new AIT treatment. It included 27 questions, taking 7–8 min to be completed (find detailed information for DQ and PQ is provided in Caimmi D et al^12^). Questions are presented in a fixed order and most of them have a close-ended format having a drop-down list of answers, aiming to avoid open answers. The 2 online questionnaires were developed according to the “checklist for reporting results of internet e-surveys, CHERRIES”.^13^

### AIT products

This survey was not intended to study any specific medication or any investigational medicinal product. No AIT product identification was recorded. Therefore, pharmacovigilance data were not collected or reported.

### Database

The SurveyMonkey® online instrument was used, allowing participating doctors to store all data on a centralized electronic database. Data gathered was anonymous and there was no traceability to the doctor or patient. The SurveyMonkey® facility operates with Secure Sockets Layer (SSL) technology, protecting user information by using both server authentication and data encryption, ensuring that user data are safe, secure, and available only to authorized persons.

### Ethics framework

The project was evaluated and approved in each country by their Ethical Committees (France: 2019_IRB-MTP_09-02; Spain: FCO–CHO-19). The CHOICE project was registered on Clinical Trials (NCT04038268). Patients were provided written informed consent. No patients nor participating doctors received any remuneration for their study-participation.

### Statistics

A sample size estimation was calculated with a minimum of 400 patients for an error lower than 5% per country. Chi square and Fisher exact tests were used to compare qualitative variables. A statistical significance of 0.05 for *p-values* was stablished. Multivariate analysis and unsupervised cluster analysis were carried out using SPAD. N^14^ software following a mixed strategy that combines divisive and agglomerative techniques. Cluster analysis considered the 13 pre-established prescription criteria to create the clusters. Characteristics of both, doctors and patients, in each cluster were further described. Statistical analysis was performed by an independent third-party using IBM SPSS Statistics, Version 25 (IBM Corp).

### Allergen immunotherapy: criteria for AIT product selection

AIT product selection by participating doctors was evaluated using 13 pre-defined criteria which were grouped into the following three categories.

#### Patient's profile

1. Disease severity
2. Patient's preferences
3. Patient's desire for fewer clinic visits
4. Patient's AIT adherence

#### Clinical characteristics of the AIT product

1. AIT safety profile
2. Prescriber's AIT-product own experience
3. AIT dose response studies
4. AIT discontinuation efficacy

#### Technical characteristics of the AIT product

1. AIT biological activity
2. Cost of AIT product for the patient
3. Cost of AIT to other payers
4. Minor allergen content in AIT
5. Major allergen content in AIT

These categories were agreed upon by the project team to be ranked as i) low/medium/high relevance on a 1 to 4 scale; ii) low (1–1.9), moderate (2–2.9) and iii) high relevance (3–4). Data were represented in a heat map, in which 5 clusters of AIT prescription patterns were identified. Once the prescription patterns were clustered, all individual answers for every DQ and PQ were analysed to define common recognizable variables in each cluster.

### Working groups’ strategy and participating doctors

The survey protocol and the questionnaires were prepared by the CHOICE Executive Group who designated proactive physicians from the Spanish and French Allergy Scientific Societies to serve as National Coordinators (NC). These NCs contacted and identified local physicians prescribing AIT willing to participate who were enrolled as Participating Doctors. Two independent data managers were responsible for helping participant physicians, cleaning the database, and coordinating statistical analysis with a third party.

## Results

### Physicians’ characteristics

One hundred and three physicians (50.5% from Spain and 49.5% from France) took part in the survey and filled out the DQ; 98.1% of them were specialists in allergy. Around 42% had ≤15 years of experience, 28% between 16 and 26 years, and 30% > 26 years of experience. Physicians estimated they attended an average of 147 patients with respiratory allergy per month and that approximately 30% of them received AIT. About 45.6% of the physicians worked exclusively in public hospitals, 35% worked exclusively in private practices, and 19.4% worked in both (Table 1). A preference to prescribe SCIT over SLIT was noted in Spain but not in France (85% vs 2% of the prescriptions respectively) where SCIT is not reimbursed since 2018.^15^

**Table 1:** Explanatory variables for identified clusters.

Section	Characteristic categories	% of all cohort, n=1735	% in CLUSTER 1 (n=355)	% in CLUSTER 2 (n=293)	% in CLUSTER 3 (n=322)	% in CLUSTER 4 (n=265)	% in CLUSTER 5 (n=500)
Doctor profile	Spain	**75.0**	**83.6**	**86.0**	**59.3**		**70.2**
	France	**24.9**	**16.3**	**14.0**	**40.7**		**29.8**
	Pediatrician	**3.0**			**5.9**		
	<15 years	**55.5**	**74.4**	**61.8**			
	16-25 years	**16.8**			**23.9**		**19.8**
	>25 years	**27.6**				**35.5**	**38.0**
Center characteristics	Private	**44.3**	**20.8**	**29.0**	**59.9**	**35.8**	**64.2**
	Public	**72.8**	**85.3**			**84.5**	
	Nurse (yes)	**79.1**	**85.9**	**86.7**	**67.1**	**84.5**	**74.8**
	2 or more physicians	**78.0**	**84.8**	**84.0**		**84.5**	
	1 physician alone	**21.9**			**33.2**		**27.0**
Evaluation method	Clinical history	**98.5**		**100**		**96.2**	
	Symptom & medication diary	**82.8**	**95.2**	**96.2**	**72.4**	**74.7**	**77.2**
	Skin prick test	**97.5**	**99.1**				
	Whole extract specific IgE	**71.8**	**92.4**	**86.3**	**58.7**		**58.0**
	Component resolved diagnosis	**78.9**	**90.7**	**92.5**			**64.4**
	Spirometry	**46.5**	**54.4**	**53.2**			**36.2**
	FENO	**7.4**	**28.7**	**2.7**	**0.9**	**0.4**	**3.0**
Patient demographics	Patient > 18 y.o.	**64.8**	**73.5**	**46.1**			**69.6**
	Patient 12 to 18 y.o.	**18.2**		**29.0**			**15.2**
	Patient < 12 y.o.	**16.9**	**11.3**	**24.9**	**23.9**	**10.6**	
	Education: University	**31.6**	**41.1**	**26.6**			
	Education: Secondary	**31.4**	**26.2**		**23.6**	**44.5**	
	Education: Primary	**17.5**	**11.8**	**28.0**		**11.7**	
	Education: Commercial/Technical	**16.0**	**20.0**	**10.6**			**18.8**
	Occupation: employed	**47.3**	**52.7**	**32.1**			**52.8**
	Occupation: student	**46.0**		**64.8**		**40.2**	
Disease characteristics	Disease onset: ≤3 y.	**31.5**		**38.2**		**21.5**	
	Disease onset: 4-9 y.	**37.1**			**30.7**		
	Disease onset: ≥ 10 y.	**31.4**		**20.5**		**41.5**	
	Rhinitis: intermitent mild	**5.2**		**9.2**			
	Rhinitis: intermitent mod/severe	**18.3**		**39.9**	**10.9**	**10.9**	**14.2**
	Rhinitis: persistent mild	**16.8**	**11.3**	**11.9**	**22.7**		**21.8**
	Rhinitis: persistent mod/severe	**58.7**	**65.9**	**37.9**		**72.5**	
	Asthma: GINA 1	**19.9**		**26.3**	**15.8**		
	Asthma: GINA 2	**16.5**		**23.5**			**13.8**
	Asthma: GINA 3	**11.8**		**6.5**		**19.6**	
	Asthma: GINA 4	**3.1**			**5.6**		**1.8**
	Asthma: Well controlled	**43.0**		**55.3**	**36.3**		
	Asthma: Partly controlled	**8.4**		**3.1**	**13.6**	**14.3**	**5.8**
	Asthma: Uncontrolled	**0.3**			**0.9**		
	Pollen monosensitized	**26.6**	**35.8**				
	Pollen polysensitized	**47.7**		**67.9**			
AIT characteristics	SCIT (any)	**64.7**		**71.7**	**54.3**	**75.1**	
	SLIT (any)	**35.3**		**28.3**	**45.6**	**24.8**	
	SCIT (allergoid)	**49.9**		**66.2**	**38.8**	**60.0**	**42.6**
	SLIT (drops)	**23.8**	**11.0**	**19.1**	**36.0**	**18.5**	**30.6**
	SCIT (natural depot)	**14.7**		**5.5**			**20.8**
	SLIT (tablets)	**11.5**	**26.7**			**6.4**	**6.0**
	AIT- Mites	**45.5**	**60.8**	**21.5**			
	AIT- Grass	**38.7**	**32.4**	**64.1**		**31.3**	**34.0**
	AIT- Trees	**26.1**	**11.0**	**51.2**		**20.4**	
	AIT- Epithelia	**4.7**	**1.40**				**8.4**
	AIT- Molds	**3.7**			**7.1**		
	AIT- Weed	**2.4**	**0.6**		**4.3**		
AIT main indication and objective	Main objective: symptom reduction	**35.0**	**18.4**		**27.8**	**49.4**	**44.4**
	Main objective: post-discontinuation efficacy	**35.0**	**29.1**	**50.5**	**42.2**		**26.2**
	Main objective: lack of disease control	**17.3**	**13.0**	**12.6**			**22.6**
	Main objective: asthma prevention	**12.1**	**38.7**	**2.0**		**1.5**	**6.2**
	Main disease: rhinitis	**73.5**	**60.3**	**90.4**		**66.8**	
	Main disease: asthma	**40.0**	**45.3**	**51.5**			**29.6**
	Main disease: conjunctivitis	**16.9**	**12.1**	**32.7**			**13.2**

Each characteristic is grouped into sections, and the average value for the whole cohort is given in the first data column. For each cluster, from 1 to 5, only data that is significantly different (p<0.05) to the whole cohort mean is shown. For clarity, data not significantly differing from the whole cohort data has been left blank. Red font represents data over the mean value, and blue font is used to signal data below the mean value for all patients.

### Patients’ characteristics

**Fig. 1:**
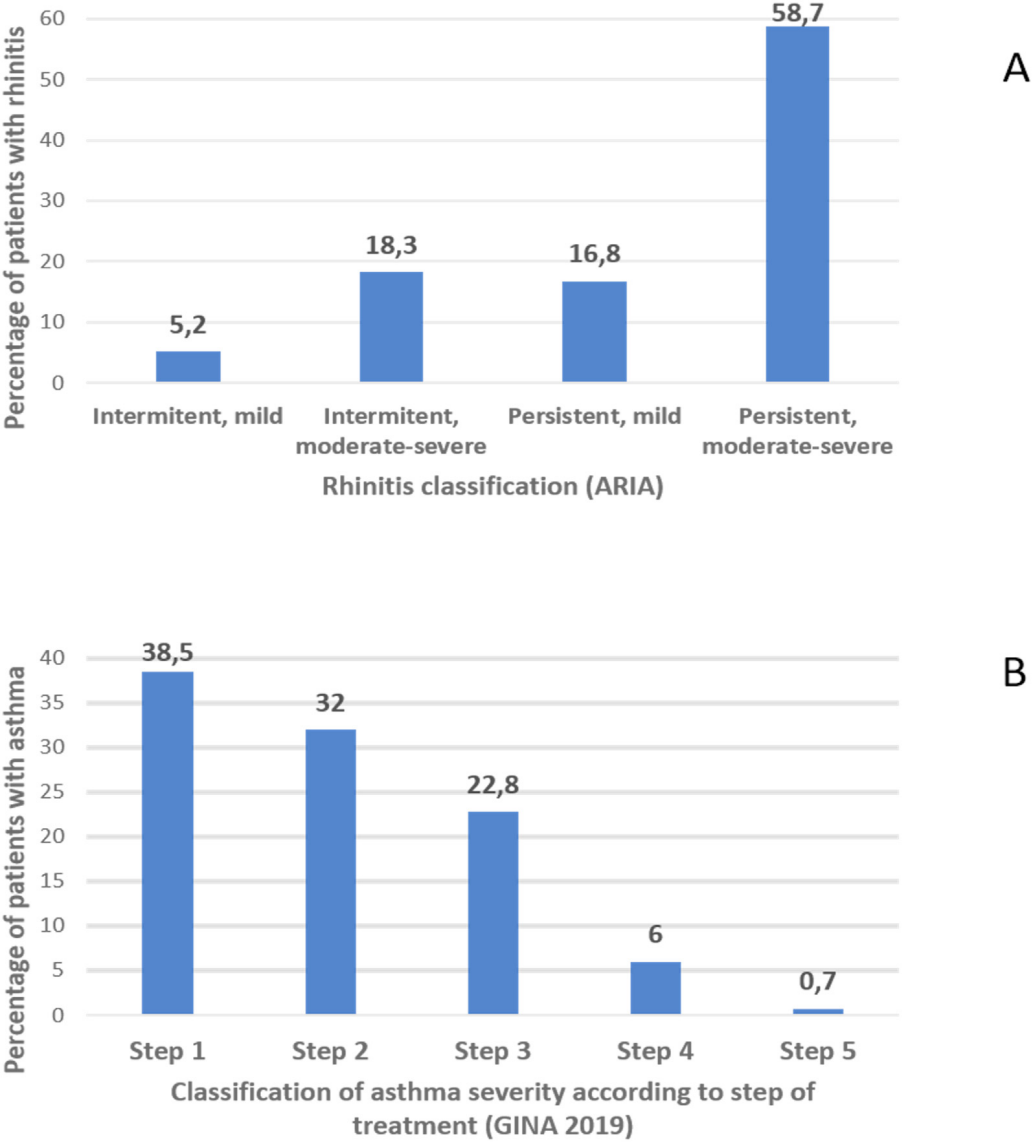
Distribution of patients with allergic rhinitis (n = 1717) according to ARIA (Allergic Rhinitis and its Impact on Asthma) guidelines (A), and distribution of patients with asthma (n = 898) according to GINA (Global Initiative for Asthma) step of treatment (B).

Data from 1735 patients (433 in France and 1302 in Spain) were recorded in the survey. Of these, 47.9% were males, and 64.8% were adults (mean age 26.2 ± SD14.2 years). The most common reported active allergic baseline diseases were allergic rhinitis (n = 1717, 99%), allergic conjunctivitis (n = 1221, 70.4%), allergic asthma (n = 898, 51.8%), atopic dermatitis (n = 241, 13.9%), and food allergy (n = 172, 9.9%). The mean time interval between respiratory allergy onset and initiation of AIT was 7.9 ± SD7.3 years. Allergic rhinitis was classified according to ARIA guidelines: 58.7% patients having moderate/severe persistent rhinitis (Fig. 1A). Asthmatic patients were classified following GINA 2019 guidelines: 70.5% were in steps 1 or 2, while only 6.7% were in steps 4 and 5 of treatment (Fig. 1B). Well controlled asthma was established for 83.1% of patients.

At inclusion, 1437 patients (82.8%) were taking 1 or more different types of symptomatic medication for their allergies: antihistamines, 76.5%; nasal corticosteroids, 51.7%; inhaled corticosteroids, 26.1%; long-acting beta agonists (LABAs), 18.6%; short-acting beta agonists (SABA), 23.5%; oral leukotriene receptor antagonists, 7.3%; oral corticosteroids, 0.6%; and biologicals, 0.1%.

### Sensitization pattern

Pollen sensitization was the most prevalent (n = 1288, 74.2%) followed by house dust mites (n = 1043, 60.1%) and animal dander (n = 694, 40%). Polysensitization to non-homologous groups, defined as positive results against at least 2 non-homologous allergen groups, was reported in 57.5% of the patients; 33.6% of the population were sensitized to 2 allergens and 23.9% were sensitized to ≥3. Sensitization to 2 or more types of pollen was observed in 64.2% of the pollen-sensitized patients. Grass pollen was the most frequently reported sensitization among pollen-sensitized patients (84.6%) followed by olive pollen (48.5%), cypress pollen (26%), plane tree (19.6%), and birch pollen (17.5%). With regard to weeds, 9.6% of the patients were sensitized to *Parietaria judaica*, and fewer than 8.4% were sensitized to mugwort.

### Diagnostic procedures used before prescribing AIT

Almost all patients were diagnosed through their clinical history (98.5%) and the results of skin prick tests (97.5%). Up to 82.8% of patients were evaluated through a symptom and medication diary. Component resolved diagnosis (CRD) was slightly more frequently used than sIgE to whole extracts (79% vs 71.8%, respectively). Spirometry was performed in 46.6% of patients (5.2% of asthmatic patients were not evaluated through spirometry) and exhaled nitric oxide was only performed in 7.4% cases. Organ specific challenge tests, such as nasal provocation tests, were used only in 1.8% of the patients.

### Allergen immunotherapy: indication, composition, schedule, and route

The main reason for prescribing AIT in these patients was AR (n = 1275, 73.5%), followed by AA (n = 695, 40.1%), and allergic conjunctivitis (n = 294, 16.9%).

**Fig. 2:**
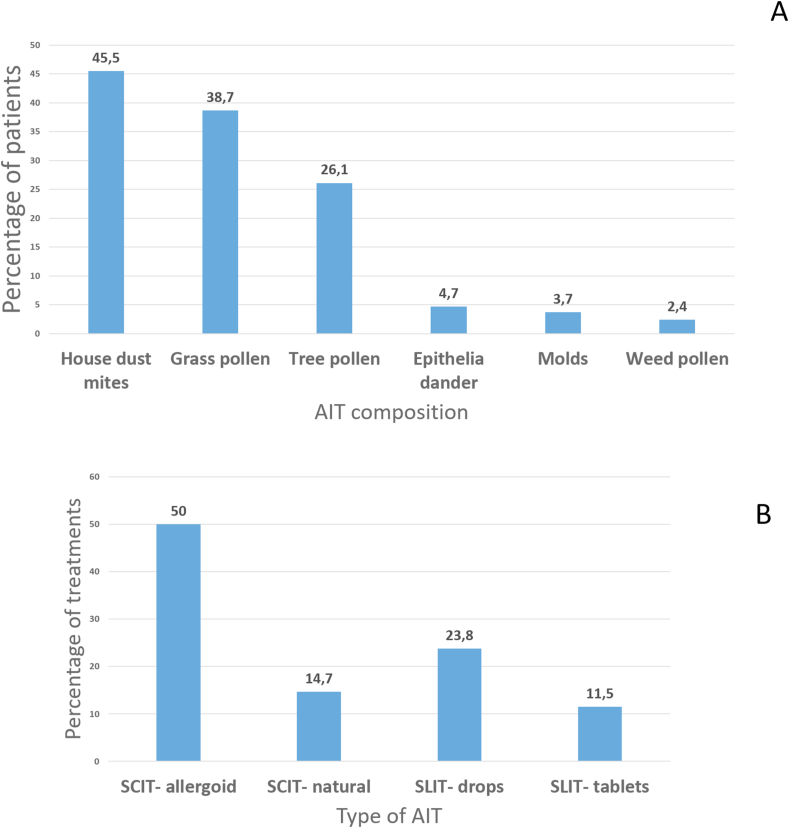
Frequency and distribution of allergen immunotherapy treatments (n = 1735) regarding their composition (A), type and route (B). SCIT, subcutaneous immunotherapy; SLIT, sublingual immunotherapy.

Overall, SCIT was more frequently prescribed than SLIT (1122 treatments, 64.7%, vs 613, 35.3%, respectively) (Fig. 2A). For SCIT, 77.3% of treatments were based on allergoid and 22.7% were based on natural extracts. For SLIT, drops (67.3%) were more commonly used than tablets (32.7%). Regarding the schedule, 77.6% were perennial while the remaining 22.4% were pre- and co-seasonal.

AIT was primarily prescribed for allergy to mites (n = 790, 45.5%), followed by grass pollen (n = 671, 38.7%), and then by tree pollen (n = 452, 26.1%), and animal epithelia (n = 82, 4.7%) (Fig. 2B).

**Fig. 3:**
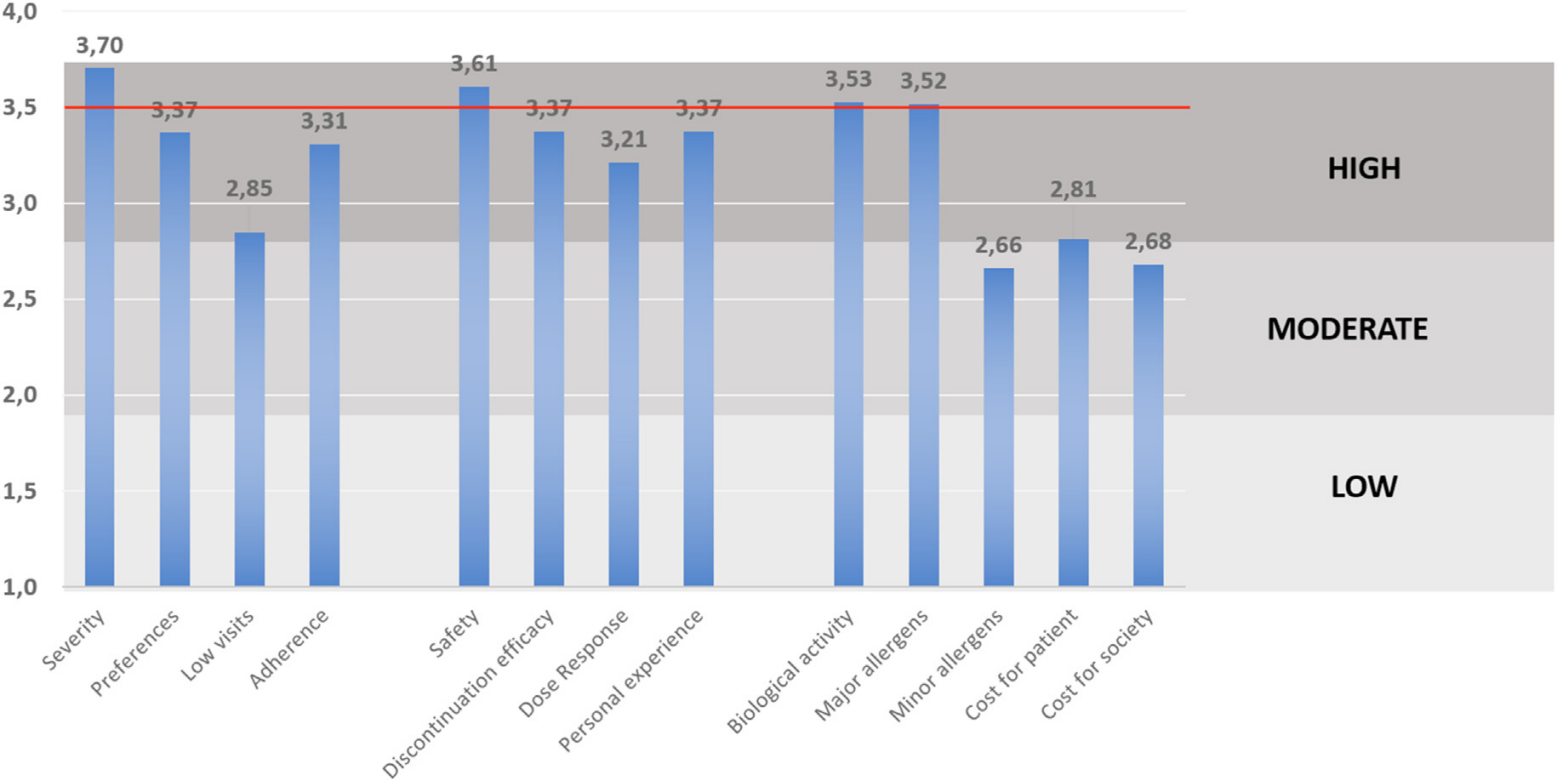
Representation of the relative clinical relevance (ranked on a scale 1 to 4) of 13 criteria investigated for every AIT prescription and grouped in three categories. Patient profile, including: severity (type and severity of the disease); preferences (patient's convenience or preferences); low visits (low number of clinical visits); and adherence (adherence or compliance concerns). Clinical characteristics of the AIT product, including: safety (documented safety profile); discontinuation efficacy (documented evidence of clinical efficacy after AIT discontinuation); dose response (documented dose response studies); and personal experience (product-specific personal clinical experience with the selected AIT product). Technical characteristics of the AIT product, including: biological activity (product-documented total biological activity); major allergens (product-documented major allergens content); minor allergens (product availability of low prevalence allergens); cost for patient; and cost for society. An arbitrary cut-off of 3.5 was established through general agreement to identify the most relevant factors for the whole sample.

As a whole, the most rated criteria for AIT selection (Fig. 3) were type and severity of the patient's disease (3.7/4), documented safety of the AIT product (3.6/4), quantification of its biological activity (3.5/4), and major allergen content (3.5/4) in the selected allergen product. Moreover, physicians took into equal consideration their personal experience with the prescribed AIT product, documented evidence of clinical efficacy after AIT discontinuation, and patient's convenience or preference (all 3 rated 3.37/4). On the contrary, the cost of the treatment seemed to be of lower relevance in terms of selecting a specific product of AIT. The lowest value was reached by the availability of minor allergens in the AIT product.

### Reasons for prescribing AIT

**Fig. 4:**
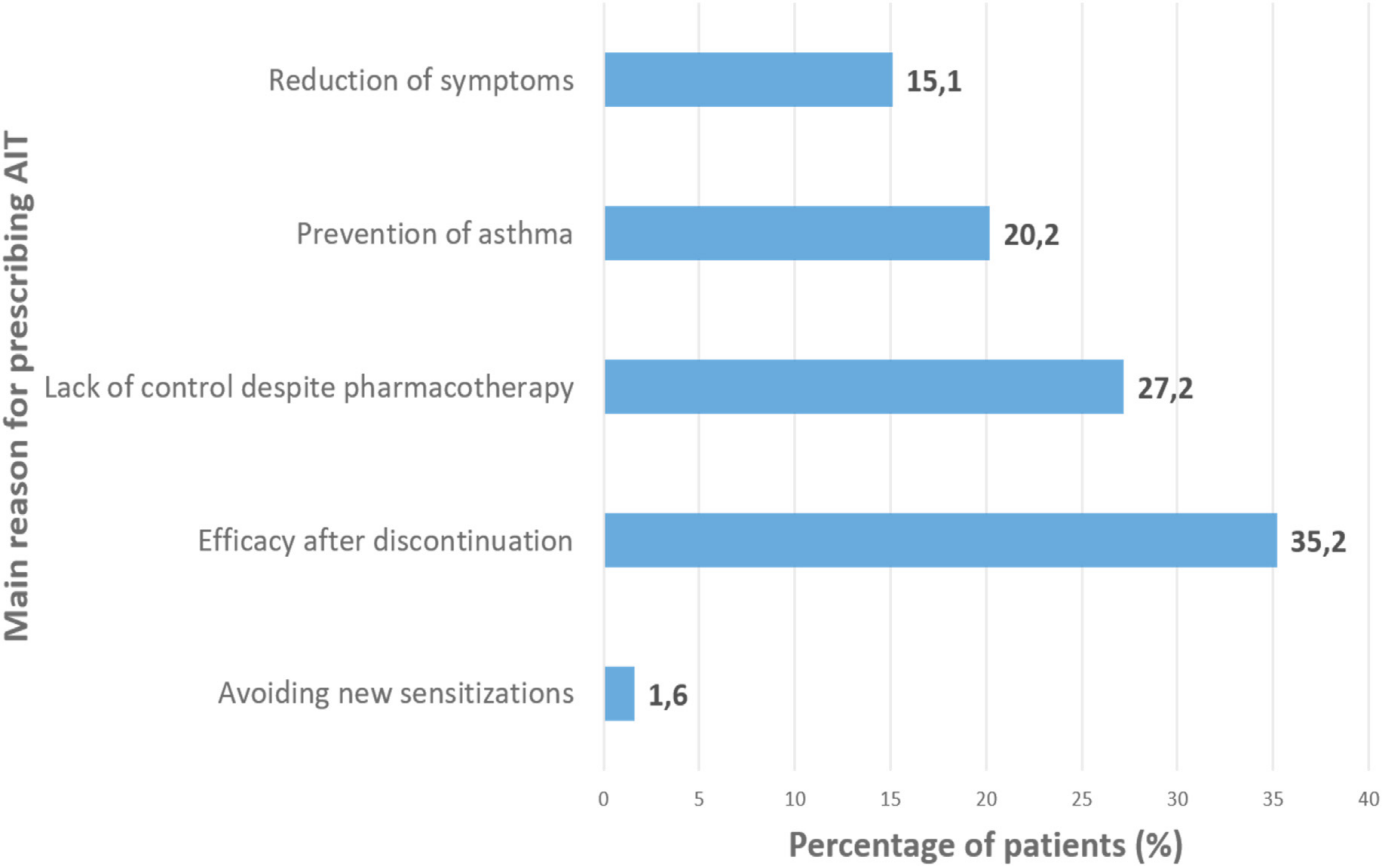
Frequency and distribution of the main reasons for AIT prescriptions in the whole sample of patients, with no respect to baseline disease.

Physicians were asked about the most relevant single reason for prescribing AIT to each patient among five different AIT indications (Fig. 4). The main reasons for prescribing AIT were: i) trying to achieve efficacy after treatment discontinuation (35.2%); and ii) the lack of disease control despite pharmacotherapy (27.2%) Avoiding new sensitizations was the least relevant reason to prescribe AIT, as reported by physicians (1.6%).

### Clusters or patterns of AIT prescriptions

• Cluster 1 - Looking at the future: asthma prevention

For those physicians who treated the 355 (20.46%) patients included in this cluster, all 13 evaluated parameters were of high relevance to prescribe AIT. Cost of treatment and presence of minor allergens content in the extract were important for these doctors, as well. Most of their patients were employed, highly educated adults suffering from moderate to severe persistent AR. Monosensitization was more frequent. HDM AIT with SLIT tablets for AA prevention was the main driver for prescription. Country analysis showed that 22.8% of the Spanish doctors would be represented in this cluster compared to 13.4% of the French doctors.

• Cluster 2: Efficacy after discontinuation of AIT with pollen

In this cluster including 293 (16.89%) patients, more attention was put on the efficacy of AIT with pollen after discontinuation. Patients were mainly children and adolescents, evaluated with the average array of allergy tests. They had a significantly shorter period between onset of the disease and initiation of AIT. They were usually pollen polysensitized patients with milder AR and well controlled AA. Patients were more frequently treated with SCIT, specifically allergoids, for tree or grass allergens. These patients received AIT with the aim of having clinical treatment efficacy over all allergic diseases, including AR, AA and allergic conjunctivitis. Country analysis showed that 19.4% of the Spanish doctors would be represented in this cluster compared to 9.5% of the French doctors.

• Cluster 3 - Fighting severe allergic disease

In this cluster including 322 (18.56%) patients, disease severity, and all characteristics of the AIT product were considered as more relevant. The efficacy of AIT after discontinuation was the most relevant factor for prescribing AIT. Patients in this cluster were less frequently evaluated for specific IgE and symptom and medication diaries. Patients mainly suffered from mild persistent AR and more severe AA (even partially controlled or uncontrolled) than those in the other clusters. The most prescribed AIT route was SLIT-drops, which was administered for less common allergens such as molds and weed. Country analysis showed that 30.3% of the French doctors would be represented by this cluster compared to 14.7% of the Spanish doctors.

• Cluster 4 - Looking at the present, facing the symptoms

Physicians treating the 265 (15.27%) patients in cluster 4 were highly selective when choosing relevant parameters as only AIT safety and biological activity were of their consideration. It is remarkable that, in this cluster, the cost of the treatment was considered as the least relevant. These patients, rarely below 12 years of age, were treated by more experienced allergists who were mainly working in public hospitals, with many healthcare workers participating to AIT activity. Symptoms control and FeNO evaluation were less frequently used. Patients tended to have a longer disease duration prior to AIT prescription and had more severe and less controlled AR and AA. There was not a predominant allergen for AIT composition. Most patients were treated with SCIT-allergoids. The main driver for prescription was the reduction of symptoms. This cluster includes French and Spanish doctors equally (12.5 vs 16.2%, respectively).

• Cluster 5 - Doctor's own clinical experience

None of the 13 factors seemed to be of high relevance for prescribing AIT to the 500 (28.82%) patients in this cluster. Physicians in this group had long experience and worked mainly in private practice. They used the fewest number of diagnostic tools before prescribing AIT. Patients were mostly adults with mild persistent AR, with less prevalence of AA or severe AA. These patients received more frequently SLIT-drops and SCIT with natural extracts. The main drivers for prescription were short term outcomes, such as reduction of symptoms and control improvement. This cluster encompasses both French and Spanish doctors with a slight predominance of the French doctors (34.4% vs 27.0%, respectively) and is, in fact, the one that most represents both (Spanish and French).

**Fig. 5:**
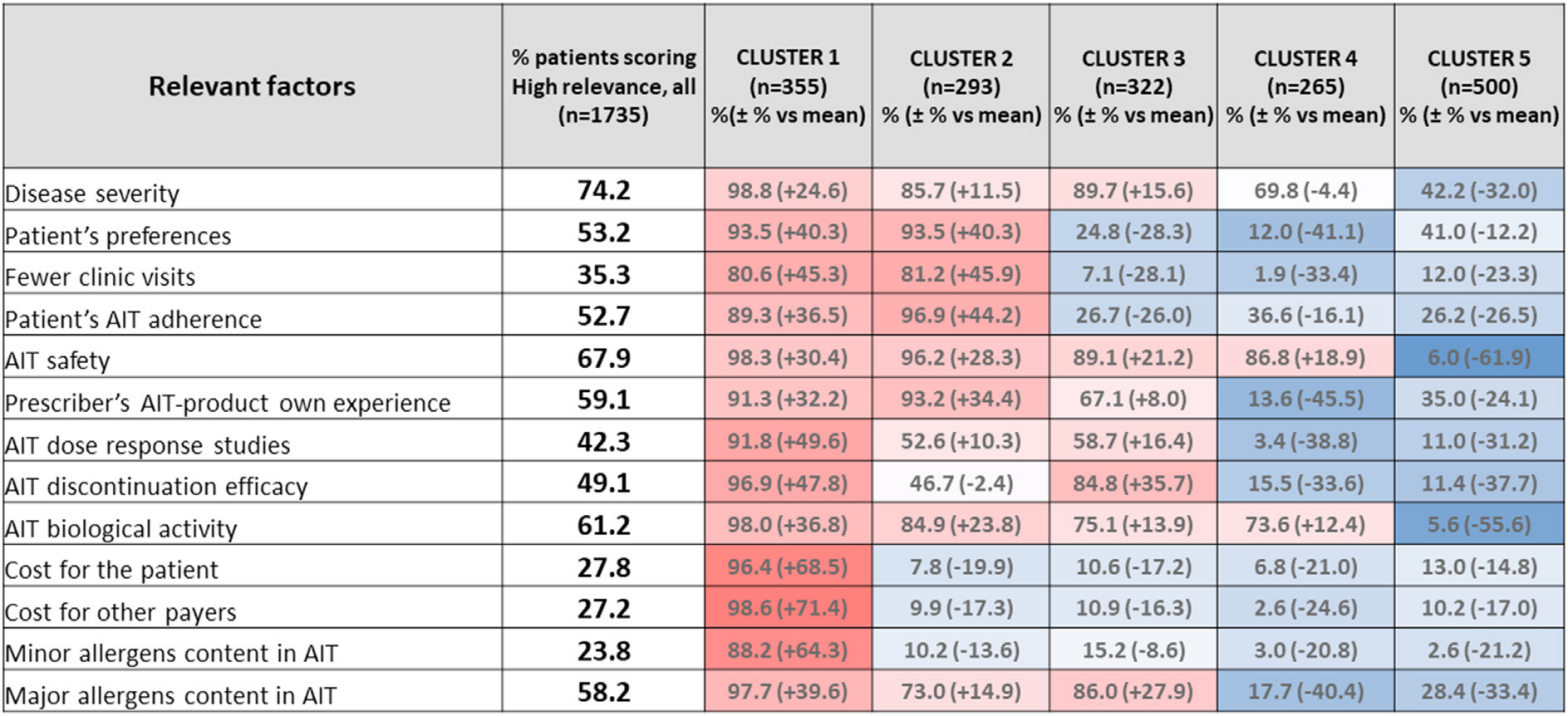
Heat map and cluster analysis built with the information gathered with the 13 clinical criteria that influenced the selection of the AIT product in each patient. All data from the DQ and PQ questionnaires were included in the analysis. The percentage of patients with highest relevance (ranked 3–4) for every factor is presented in the first column. The default colour gradient sets the lowest value in the heat map to dark blue, the highest value to a bright red, and mid-range values to light blue or red, with a corresponding transition (or gradient) between these extremes. Thus, each coloured cell on the map corresponds to a concentration value and the intensity of the colour is directly related to the difference with respect to the value in the whole sample. According to the combinations of colours, five clusters were displayed.

A detailed information on the cluster analysis and its interpretation are shown in Table 1, Table 2, and Fig. 5.

**Table 2:** Clusters of AIT prescription.

	CLUSTER 1 Looking at the future: asthma prevention	CLUSTER 2 Efficacy after discontinuation with pollen	CLUSTER 3 Fighting severe allergic disease	CLUSTER 4 Looking at the present: facing the symptoms	CLUSTER 5 Doctor's own clinical experience
	*Asthma prevention, after highly considering all parameters of relevance for prescriptionOpposed to cluster 5*	*Persistence of efficacy after grass/tree AIT, following patients' preferences and complianceOpposed to cluster 4*	*Persistence of efficacy after AIT, even in severe asthma patients, treated also for less common allergens*	*Symptoms reduction with a focus on asthma, regardless of other symptoms and patients' preferencesOpposed to cluster 2*	*Symptoms' control and reduction, especially for allergic rhinitisOpposed to cluster 1*
**Prescribers**	Younger allergists, mainly working in public hospitals, with numerous staff, and a significant use of evaluation methods for whom AIT prescription needs to be based on all 13 parameters of relevance, with a particular focus on the cost (both for patients and for other payers). More frequently in Spanish doctors.	Young allergists, giving a lot of relevance to patients' preferences and treatment compliance. More frequently in Spanish doctors.	Allergists, mainly working in private practices, who pay attention to major allergens and to the efficacy of AIT after treatment discontinuation. More frequently in French doctors.	More experienced allergists, mainly working in public hospitals, not paying attention to patients' preferences, nor to their own experience, nor to the content in major allergens of the products. No differences regarding country.	More experienced allergists, mainly working in private practices, who don't consider any of the 13 parameters of relevance, especially they don't consider neither the product's safety, nor its biological activity. More frequently in French doctors.
**Patients**	Mainly adult patients who undergo a complete allergy work-up prior to AIT prescription, monosensitized and suffering from severe persistent allergic rhinitis.	Pediatric and adolescence patients, mainly students, that undergo a complete allergy work-up prior to AIT prescription, with a special focus on biologic evaluation of IgE and CRD and suffering from intermittent or persistent moderate to severe allergic rhino-conjunctivitis or controlled mild asthma. Polysensitized with recent disease onset	Equally pediatric and adult patients, less frequently evaluated by whole extract specific IgE, and mainly suffering from persistent allergic rhinitis, and more severe asthma than in the other groups (even partially controlled or uncontrolled).	Mainly adult patients, whose clinical history and symptoms are less considered during the allergy work-up, suffering from moderate to severe persistent allergic rhinitis or moderate persistent, even partially controlled asthma.	Mainly adult patients, for whom diagnosis is mainly based on clinical history and symptoms, mainly suffering from persistent allergic rhinitis, and less asthmatic than in other groups
**AIT**	Mostly treated for HDM allergy, and with more tablets than the other groups, specifically prescribed to prevent asthma.	Mostly treated with SCIT for grass or trees allergy, looking for treatment efficacy after AIT discontinuation.	Mainly SLIT by drops, with more patients treated for molds and weed than in the other groups, and with the main goal of treatment efficacy after AIT discontinuation, and no regard to symptoms reduction during treatment.	Mostly treated with SCIT, to reduce symptoms, especially asthma-related ones.	More prescription for epithelia, with both SCIT and SLIT by drops as well, to reduce symptoms and improve control.

## Discussion

Our study offers a novel independent academic international survey and cluster analysis to understand prescription drivers of AIT in real life, with no bias related to the AIT product names nor manufacturers. In this survey, doctors voluntarily participated, unpaid, motivated only by their altruistic will to perform research. Doctors' and patient's anonymity was protected throughout the survey, which gave more confidence to participants to share their true opinions. We identified 5 different prescription patterns: avoiding asthma onset, prioritizing patient's preferences, focusing on severe disease, aiming at symptom control, and relating to prescriber's personal experience. These different patterns of prescription are not a unique model of a prescriber and specific patient, but the result of a multilayer interaction of several factors that we are only starting to understand.

We collected real-life information from 1735 patients, 99% had AR, 70.4% had allergic conjunctivitis and 51.8% had AA. Asthma alone was only present in 0.9%, reflecting that AA without AR is either infrequent or just a minor determinant in AIT prescription, as was also described by Migueres et al^16^ in a transversal study where only 2% patients had asthma alone. In most cases, AIT prescriptions followed international guidelines. Indeed, clinical AR guidelines^8^ recommend AIT only in case of intermittent moderate-to-severe AR, or persistent AR of any severity (observed in 94.8% of the evaluated population), resisting conventional symptomatic drugs (83% of patients were on pharmacotherapy). Most asthmatic patients had mild to moderate asthma (93.3% steps 1, 2, and 3 according to GINA^9^) and well-controlled symptoms (83.1%, which is in line with the precautions on the use of AIT in patients suffering from asthma).^17^ Although not mandatory in current guidelines, 80% of patients had a molecular diagnosis done, as recommended by novel AIT prescription algorithms.^18^

CHOICE corroborated a strong country-dependent selection of AIT route (98% SLIT in France and 85% SCIT in Spain), probably explained by differences in medical education programmes, access to AIT products, and AIT reimbursement policies.^15^^,^^19^

Considering the 13 predefined critical factors influencing AIT prescription (Fig. 3), baseline disease severity was the most relevant driver, in line with current guidelines. Although AIT is extremely safe,^7^^,^^20^ prescribers are giving more relevance to this aspect than to efficacy parameters. Patient's preferences, adherence, and, more markedly, number of visits or AIT cost for the patient, although key factors influencing precision medicine,^21^ are poorly considered by prescribers.

When participants were requested to select only 1 (Fig. 4) main reason to prescribe AIT, efficacy after discontinuation was the leading cause in one third of the patients, which is partially surprising due to the weaker evidence supporting this outcome compared to AIT efficacy to reduce symptoms^1^ or prevent asthma.^20^^,^^22^ However, symptom reduction while on use and asthma prevention were ranked second and third as proposed by guidelines^8^ and previous surveys.^23^ Strikingly, asthma prevention was the main goal of 38.1% of Cluster 1 prescriptions (Table 1) while only 26.5% of patients were below 18 years old, providing novel views of a real-life use of AIT beyond evidence-based data, as this effect has only been proven in younger populations.^22^ Prevention of new sensitizations was almost neglected as a prescription driver, which is in accordance with other published data.^22^^,^^24^

The use of cluster and latent class analysis is increasing in the field of allergy due to its validity to analyse large datasets.^25^^,^^26^ This tool highlighted in CHOICE that there is no uniform indication of AIT and allowed us to segregate 5 different clusters of analysis, with cluster 1 and 2 being more representative of the practice in Spain, and cluster 3 and 5 reflecting practice in France.

Clusters 1 and 5 represent two opposite ways to carry out AIT prescription. Cluster 1 represents a group of younger allergists, giving high relevance to all 13 predefined drivers (Fig. 5), showing an extensive use of all diagnostic methods in highly staffed centres, prescribing last generation AIT (higher prescription of tablets), and focusing on asthma prevention (thus called “looking at the future, aiming to prevent asthma”). Cluster 5 is the largest cohort of patients in which doctors, mainly working in private practice with less personnel, are giving lower relevance to all 13 predefined drivers for AIT prescription. They base their decision criteria on their own clinical experience (thus called “doctor's own clinical experience”). They use more SCIT and SLIT-drops to merely treat uncontrolled rhinitis for short term symptom reduction.

Cluster 2 focused on polysensitized children and adolescents with milder AR and well-controlled asthma. SCIT was the preferred route for AIT administration, which is a little bit surprising, considering both that this cluster included mainly a pediatric population and that AIT evidence for that population is better for SLIT than for SCIT.^1^^,^^27^ This group aims to see treatment efficacy in reducing current symptoms and also to achieve efficacy after treatment discontinuation (thus named “efficacy after AIT discontinuation”); doctors understand that respiratory allergy is a chronic disease which means life-long pharmacotherapy, and they know that most patients want a “cure” or at least something allowing them to use less symptomatic medication.

In Cluster 3, patients had more severe asthma and doctors put more emphasis on disease severity (therefore called “fighting severe allergic disease”) and medication safety. Severity has been described to be a major AIT prescription trigger also in a previous report on doctor's preferences in an Italian cohort.^28^ As in Cluster 2, the reduction of symptomatic medication was an important outcome to consider when prescribing AIT.

In cluster 4, patients had longer duration of symptoms and less controlled allergic disease; doctors had more years of clinical experience, worked mainly in public hospitals, and favoured SCIT to address the severity of the symptoms (thus called “looking at the present, facing the symptoms”).

We are aware of the inter-country differences and the difficulties in interpreting results from the 2 heterogeneous groups, doctors and patients with different socio-economical, cultural and demographic characteristics; however, to identify drivers of AIT prescription we needed to have a large cohort of individuals, all of them with the same criteria: “receiving AIT as new treatment for their respiratory allergic condition” and the methodology chosen allowed to include all data in the same analysis and overcome the heterogeneity issue. However, the cluster analysis carried out has proven to be effective as it allowed the identification of “clusters” that would represent doctors’ patterns of prescription from both countries, this was possible due to the large size of the sample analysed.

In conclusion, the CHOICE study highlights the reasons and patterns of AIT prescriptions in real-life clinical settings. It provides new insights in the complex decision-making process of AIT prescription, identifying different drivers, without addressing whether several of these profiles are associated to better or worse efficacy results. While some doctors used all 13 criteria proposed in our survey, others just ignored them. Maybe this is due to years of experience, confidence, healthcare system, local resources, or to the fact that our criteria were missing other relevant drivers, and probably, deeper qualitative analyses on clusters 4 and 5 may reveal new prescription drivers. CHOICE survey is currently under development in some other regions in the world (CHOICE-GLOBAL). Due to time differences related to ethical approval time, recruitment of participating doctors and the SARS-CoV-2 pandemic, not all data have been collected at the same time. CHOICE results will be determinant to understand regional differences on the topic, which will be of high value to improve healthcare pathways, policy making and educational programs regarding AIT.

## Financial Support

No economical compensation was paid to any Survey's Doctor or Survey's Patient participant. CHOICE project for France and Spain was supported by an unrestricted Educational Grant provided by ALK and Stallergenes-Greer. Sponsor do not had any decision taking in the study design, the collection, analysis and interpretation of data; in the writing of the report, nor in the decision to submit this article for publication.

## Data availability statement

Data can be available on reasonable justified request to the corresponding author.

## Author Contributions

PRdR, DC, PRN, CV, CM, PD, and MC were responsible for the design of the study, asking for permissions, searching for participating doctors, following the survey analysing data and writing the manuscript.

MTGF, MTP, AB, IB, and HBT were the doctors from Spain and France who had recruited more patients.

## TBC supervised the whole study

All authors have made substantial contributions for the conception and design of the study, the acquisition of data, the analysis and interpretation of data and drafted and/or revised it critically providing their approval to the final version.

## Ethics approval

The project was evaluated and approved in each country by their Ethical Committees (France: 2019_IRB-MTP_09-02; Spain: FCO–CHO-19). The CHOICE project was registered on Clinical Trials (NCT04038268). Patients were provided written informed consent. No patients nor participating doctors received any remuneration for their study-participation.

## Conflicts of interest

Pablo Rodriguez del Rio has received honorarium as speaker from Stallergenes Greer, ThermoFisher Scientific, Leti, GSK, DBV, Aimmune Therapeutics, DBV, FAES, Miravo and Sanofi.

Davide Caimmi has received honorarium as a consultant and speaker from ALK, Stallergenes Greer, AstraZeneca, Aimmune, Mylann, and Sanofi.

Pilar Rico Nieto declares no conflicts of interest.

Carmen Vidal has received honorarium as speaker from ALK, Stallergenes Greer, AstraZeneca, ThermoFisherScientific, Leti, Hal-Allergy, Chiesi, Mundipharma, GSK, and Sanofi ouside the submitted work.

Carmen Moreno declares no conflicts of interest related to this manuscript.

Gonzalez-Fernández MT declares no conflicts of interest.

Tomás-Pérez M declares no conflicts of interest.

Beristain A: declares no conflicts of interest.

Bosse I: declares no conflicts of interest.

Trinh HB: has received honorarium as a speaker from ALK, Stallergenes Greer, Menarini, Viatris, AstraZeneca.

Thomas B. Casale reports consulting fees from Thermo Fisher Scientific, Genentech, Novartis, outside the submitted work.

Pascal Demoly has received fundings from ALK, Stallergenes Greer, AstraZeneca, ThermoFisherScientific, Ménarini, GSK, Zambon, Viatris, Puressentiels through his institutions.

Moises A. Calderon has received honorarium as consultant and speaker for ALK.

## Consent of Authors

All authors gave their consent for publication in World Allergy Organization Journal.

## References

[1] Roberts G., Pfaar O., Akdis C.A. (2018). EAACI guidelines on allergen immunotherapy: allergic rhinoconjunctivitis. Allergy.

[2] Alvaro-Lozano M., Akdis C.A., Akdis M. (2020). EAACI allergen immunotherapy user’s guide. Pediatr Allergy Immunol.

[3] Agache I., Lau S., Akdis C.A. (2019). EAACI Guidelines on Allergen Immunotherapy: house dust mite-driven allergic asthma. Allergy.

[4] Wahn U., Bachert C., Heinrich J., Richter H., Zielen S. (2019). Real-world benefits of allergen immunotherapy for birch pollen-associated allergic rhinitis and asthma. Allergy.

[5] Zielen S., Devillier P., Heinrich J., Richter H., Wahn U. (2018). Sublingual immunotherapy provides long-term relief in allergic rhinitis and reduces the risk of asthma: a retrospective, real-world database analysis. Allergy.

[6] Devillier P., Molimard M., Ansolabehere X. (2019). Immunotherapy with grass pollen tablets reduces medication dispensing for allergic rhinitis and asthma: a retrospective database study in France. Allergy.

[7] Calderón M.A., Vidal C., Rodríguez Del Río P. (2017). European survey on adverse systemic reactions in allergen immunotherapy (EASSI): a real-life clinical assessment. Allergy.

[8] Bousquet J., Schünemann H.J., Samolinski B. (2012). Allergic rhinitis and its impact on asthma (ARIA): achievements in 10 years and future needs. J Allergy Clin Immunol.

[9] Global Initiative for Asthma [Internet]. Global initiative for asthma - GINA. [citado 18 de agosto de 2016]. Disponible en: http://ginasthma.org/.

[10] Canonica G.W., Bachert C., Hellings P. (2015). Allergen immunotherapy (AIT): a prototype of precision medicine. World Allergy Organ J.

[11] Incorvaia C., Al-Ahmad M., Ansotegui I.J. (2021). Personalized medicine for allergy treatment: allergen immunotherapy still a unique and unmatched model. Allergy.

[12] Caimmi D., Rodríguez del Río P., Rico P. (2023). Criteria used by health professionals on the selection of allergen immunotherapy in real clinical practice: methodology. World Allergy Org J.

[13] Eysenbach G. (2004). Improving the quality of web surveys: the checklist for reporting results of internet E-surveys (CHERRIES). J Med Internet Res.

[14] SPAD [Internet]. Système Portable pour l'Analyse des Données. [citado 10 de febrero de 2023]. Disponible en: https://ia-data-analytics.com/.

[15] Caimmi Davide E., Demoly P. (2019). Allergen immunotherapy using NPP: perspectives for the treatment and prevention of respiratory allergies; the case of pollinosis. Rev Fr Allergol.

[16] Migueres M., Fontaine J.F., Haddad T. (2011). Characteristics of patients with respiratory allergy in France and factors influencing immunotherapy prescription: a prospective observational study (REALIS). Int J Immunopathol Pharmacol.

[17] Pitsios C., Demoly P., Bilò M.B. (2015). Clinical contraindications to allergen immunotherapy: an EAACI position paper. Allergy.

[18] Barber D., Diaz-Perales A., Escribese M.M. (2021). Molecular allergology and its impact in specific allergy diagnosis and therapy. Allergy.

[19] Paoletti G., Di Bona D., Chu D.K. (2021). Allergen immunotherapy: the growing role of observational and randomized trial «Real-World Evidence. Allergy.

[20] Fritzsching B., Contoli M., Porsbjerg C., Buchs S., Larsen J.R., Freemantle N. (2022). Real-world evidence: methods for assessing long-term health and effectiveness of allergy immunotherapy. J Allergy Clin Immunol.

[21] Hellings P.W., Fokkens W.J., Bachert C. (2017). Positioning the principles of precision medicine in care pathways for allergic rhinitis and chronic rhinosinusitis - a EUFOREA-ARIA-EPOS-AIRWAYS ICP statement. Allergy.

[22] Halken S., Larenas-Linnemann D., Roberts G. (2017). EAACI guidelines on allergen immunotherapy: prevention of allergy. Pediatr Allergy Immunol.

[23] Leatherman B., Skoner D.P., Hadley J.A. (2014). The Allergies, Immunotherapy, and RhinoconjunctivitiS (AIRS) survey: provider practices and beliefs about allergen immunotherapy. Int Forum Allergy Rhinol.

[24] Di Lorenzo G., Leto-Barone M.S., La Piana S., Plaia A., Di Bona D. (2017). The effect of allergen immunotherapy in the onset of new sensitizations: a meta-analysis. Int Forum Allergy Rhinol.

[25] Fitzpatrick A.M., Bacharier L.B., Jackson D.J. (2020). Heterogeneity of mild to moderate persistent asthma in children: confirmation by latent class analysis and association with 1-year outcomes. J Allergy Clin Immunol Pract.

[26] Mastrorilli C., Tripodi S., Caffarelli C. (2016). Endotypes of pollen-food syndrome in children with seasonal allergic rhinoconjunctivitis: a molecular classification. Allergy.

[27] Dhami S., Nurmatov U., Arasi S. (2017). Allergen immunotherapy for allergic rhinoconjunctivitis: a systematic review and meta-analysis. Allergy.

[28] Larosa M., Ciprandi G., Tesi C.F. (2009). Specific immunotherapy for allergic rhinitis in Italy: the doctors points of view. Int J Immunopathol Pharmacol.

